# Guy's Hospital Reports

**Published:** 1843-10

**Authors:** 


### Art. II.

*Guy's Hospital Reports.* Edited by G. H. Barlow, m.d. and J. P. Babington, m.a. Vol. VII. (Nos. XIV-XV.) — *London*, 1842-3. 8vo, pp. 496.

In the throng of other candidates clamorous for notice, we have allowed our old friends of Guy's to get into the rear rank ; we now proceed to do justice to the Reports in a brief analysis, according to our wont.

1. *Cases illustrative of the diagnosis of disease of the kidney, by* Dr. Barlow. The main objects of this paper are—first, to show the value of sickness, or rather irritability of the stomach, as a distinguishing symptom between diseases of the kidneys, accompanied with irritation of those organs, and affections of other structures in the neighbourhood ; and secondly, to add to the stock of information already possessed regarding the peculiar cerebral affections dependent upon the non-depuration of the blood by the kidney. It deserves an attentive perusal, for the cases noticed belong to a set which often perplex the most accomplished physician.

II. This is another admirable communication from the pen of Mr. TAYLOR, entitled *Medico-legal report of a case of infanticide, with additional remarks on the fetal lungs.* Our attention must be confined to the latter part, which forms the continuation of a former paper, already fully analysed in this Journal, (Brit. and For. Med. Rev. vol. VI. p. 62.) In that article some examinations of fetal lungs were reported, and certain inferences deduced from them. The present contains the particulars of fourteen additional cases, some observed by Mr. Taylor himself, and others communicated. The results to which these investigations have led him will be seen by the following abstract:

1. *Of the test of Ploucquet.* Mr. Taylor is now of opinion "that the test is not even capable of furnishing corroborative proof, and that it would be unsafe to rely upon it." It will be evident from the annexed table that there is no sort of constant relation between the weight of the lungs and that of the body, and that when the body is below the average, it by no means necessarily follows that the lungs should be in the same condition:

*Before Respiration.*

| | Weight of the body. | Lungs. | Ratio. |
|---|---|---|---|
| I. | 57,000 gr. | 694 gr. | 1 : 82 |
| II. | 62,660 ,, | 683 ,, | 1 : 91 |
| III. | 34,540 ,, | 630 ,, | 1 : 54 |
| IV. | 47,170 ,, | 703 ,, | 1 : 67 |
| V. | 51,890 ,, | 744 ,, | 1 : 70 |
| VI. | 29,460 ,, | 520 ,, | 1 : 57 |
| VII. | 29,966 ,, | 666 ,, | 1 : 45 |
| VIII. | 47,025 ,, | 658 ,, | 1 : 71 |
| IX. | 39,370 ,, | 550 ,, | 1 : 71 |

Compare with this the following table, exhibiting the ratios obtained from lungs in which the process of respiration had been established:

*After Respiration.*

| | Weight of the body. | Lungs. | Ratio. |
|---|---|---|---|
| I. | 56,160 gr. | 1000 gr. | 1 : 56 |
| II. | 34,125 ,, | 861 ,, | 1 : 39 |
| III. | 41,788 ,, | 920 ,, | 1 : 45 |

It should be observed that in case II. respiration was imperfect, though the child lived some days.

2. *Absolute weight of the lungs.* In the former paper the mean weight of healthy lungs, from six mature children which had died *without breathing*, was stated to be 569 gr., in the nine cases reported now, the average weight was 649 gr. Mr. Taylor correctly observes, that it is of the utmost importance that the maturity of the child should be fully ascertained in making estimates of this kind, and it would, therefore, be well that the reporters, instead of resting satisfied with the bare assertion that such was the case, should describe the general characters presented, by which means others would be enabled to judge for themselves.

The average weight of the lungs *after respiration*, derived from the three cases in the table, is 927 gr., but the degree to which respiration has been established must materially influence the result. There is also another source of fallacy which, to our minds at least, would render all deductions from experiments of this kind of very small value, viz. the impossibility of determining the previous weight of the organs, without which it is clear that even an approach to certainty cannot be anticipated; inordinately small lungs, even when fully dilated, may not reach the common average. In respect of immature children, Mr. Taylor remarks that there would be great risk in giving an opinion of respiration having taken place from the state of the lungs. Cases xiii. and xiv. in this paper illustrate the observation. They were both seven-months' children. In case xiii. the lungs weighed 559 gr.; the child lived an hour, respiring, but with difficulty. The organs, even when divided into 48 pieces, sank in water. In case xiv. the lungs weighed 556 gr.; the child lived thirty-six hours, breathing freely, and the lungs floated even when divided into 60 pieces. Here the difference of weight only amounted to three grains, and yet the lungs of one were filled with air, while not a trace of it could be detected in those of the other child. Upon the whole, then, it is evident that the question is one to which a conclusive answer cannot easily be found.

3. *Artificial inflation.* The experiments related in this paper are, for the most part, confirmatory of the opinion formerly expressed, that artificially inflated lungs, as well as those in which natural respiration has been carried on to a limited extent only, may be deprived of the contained air by pressure; but they show also that much greater force is sometimes required than has been generally supposed. Where the lungs are heavy, have great buoyancy, and cannot be made to sink by firm compression, Mr. Taylor thinks we have good corroborative proof of the child having breathed. Considerable weight alone is of little value.

4. *Signs of maturity.* The cases reported in this paper show that in mature children the umbilicus is situated from a quarter to half an inch below the centre of the body, instead of exactly coinciding with that point, as Chaussier has stated.

5. *Signs of live birth.* Mr. Taylor's observations have confirmed the opinion that the circle of redness at the junction of the umbilical cord, with the skin of the abdomen, is no proof of live birth. It was found in five cases of stillborn children, and was absent in others which had survived birth. Dr. Geoghegan observed a slight contraction about the centre of the ductus arteriosus in two children which were born dead. This point requires further investigation.

We need scarcely say that we strongly recommend this paper to the notice of our readers.

iii. *Observations on pelvic tumours obstructing parturition, by* John C. W. Lever, Esq. This is a very lengthened communication, extending to sixty-eight pages. A brief abstract would be of little value, and we must therefore pass it by in silence. To this course we are the less disinclined from the fact that, although containing a good account of the diseases in question, and exceedingly judicious rules for their treatment, the author has not propounded any views beyond those which are generally received and acted upon by the profession.

iv. *On the digestive solution of the œsophagus, and on the distinct properties of the two ends of the stomach, by* Mr. T. Wilkinson King. That the coats of the stomach are not unfrequently dissolved and perforated, to a greater or less extent, by the action of the gastric fluid after death, is a fact now almost universally recognized, though opinions are still divided as to the necessity of previous disease in the membrane to enable this process to be set up. This point is not specifically noticed by our author, but we should judge that he would answer the question by a negative, for he says that "in the autopsies in London the effects of digestion on the stomach itself are *as often discoverable as not;*" and it is scarcely probable that in half the cases of death there should be affections of that viscus. We apprehend, however, that the experience of other practitioners will not show so great a proportion; we have opened a considerable number of bodies in our time, and been present at the examination of many more, and certainly never met with anything like so great a number. Mr. King observes "it is to be regretted that the inferences afforded by these effects have not had much more influence in limiting and directing the objects of experimentalists on the subject of digestion. When, for instance, the coats of the stomach have been perforated, and the surface of the spleen has become similarly dissolved or the diaphragm perforated, is it not evident that the agencies of the par vagum and of peristaltic motion are altogether inadmissible, as essentials to the solvent action?" This is perfectly true, if the mere *solvent* power of the fluid be regarded, and the same is abundantly proved by the experiments on artificial digestion *out of the body;* but we take it, the question to be determined (and it does not appear to us to have ever yet been satisfactorily cleared up), is not this, but—What influence have the par vagum and other nerves upon the *entire process of digestion?* including under this head the secretion of the gastric juice, as well as its influence upon the alimentary matters contained in the stomach, when once effused. As far as our own observations extend, we feel convinced that these parts have a most important office to perform, in whatever way their action may be explained. But upon this discussion we cannot enter.

Solutions of the œsophagus are also common, but not to such an extent as in the two cases narrated by Mr. King. The first of these is an example of a very extreme kind; the whole circumference of the tube was destroyed in two places, just above the stomach, an intermediate portion about an inch in length being unaffected, probably from the weight of the heart dislodging the fluid. The edges of the divisions were soft and flocculent. The fluid had escaped into the right pleural sac, and the investing membrane of the obtuse posterior edge of the lung was entirely destroyed, over a space of about twelve square inches, the alteration being circumscribed by an abrupt margin of flocculent pleura, beyond which the serous membrane bore a natural appearance. The stomach was entire. Mr. King believes that the portion of the tube immediately above the cardiac sphincter is most commonly affected.

The remainder of the paper is occupied with a series of observations in support of the opinion that the left half of the stomach is alone concerned in the production of the digestive fluid. After noticing the testimony of various authors to this fact, and the frequent existence of a line of demarcation, passing perpendicularly across the lining membrane of the viscus, a little to the right of the œsophageal opening, and on the pyloric side of which he believes solution never takes place, (excepting from accidental circumstances, such as the casual passage of the proper secretion of the left cul-de-sac, from position or other causes,) the author proceeds to narrate some experiments of a rather novel character. They consisted in the testing of the two ends of the stomach, previously well washed with litmus paper, and the result was almost invariably the same; the fragments of paper laid on the cul-de-sac exhibited the presence of an acid, while those which were in contact with the pyloric half were either altogether unchanged or but feebly reddened.

We cannot regard these experiments as by any means conclusive. They are exposed to many sources of fallacy, more especially from the influence of gravitation, which would necessarily cause any fluid, that might remain in the stomach at the time of death, to collect in the great end of the organ; and although this were removed, and the fluid adhering to the surface entirely washed away, still a sufficient quantity would have permeated the tissues, before examination, to render the results doubtful. Case 8 affords a striking corroboration of this opinion. In this case "the stomach contained but little, and was acid at all parts, but having been well washed under the watercock, and set aside (sprinkled with litmus cuttings), for twenty-four hours, *no part* evinced acidity." The stomach was opened *four* hours after death, which would scarcely allow time for extensive permeation. Still we would not deny that there is some value in these experiments, and we shall quote the one which appears to us least liable to objections:

"Case iii, Jan. 13, 1842. Maria H., about thirty-five years of age, fat and intemperate, was found dead. The body was examined about six hours from the probable time of her decease. There was much serous effusion in the head. The liver was tumid and lacerable. The stomach contained only about an ounce of mucus, scented with gin, and not acid. The stomach, being washed under the water-cock, and sprinkled with litmus cuttings, exhibited acid re-action, in the cul-de-sac only, after twenty-four hours." (p. 152.)

Mr. King is opposed to the idea that the mucus performs any part in the solvent actions; he chiefly regards it as the protective means by which the coats of the stomach escape injury, and deprecates the idea of any other agency being required for this purpose.

v. *Two cases of injury of the head, followed by symptoms of compression, produced respectively by extravasation of blood and formation of pus, relieved by operation, by* Edward Cock. These are two interesting cases, but we have not space to give more particulars than are contained in the title of the paper.

vi. *Observations on urinary concretions and deposits, by* Golding Bird, m.d. This is a paper of considerable value, and most creditable both to the industry and chemical knowledge of the author. It is chiefly occupied with an account of the calculi in the museum of Guy's Hospital, but is interspersed with remarks derived from other sources of personal experience. The collection now contains 363 specimens, all of which, excepting 21, have been divided so as to exhibit their internal structure. The examination was not limited to the composition of the external crust, but had respect to every layer, and particularly to the chemical characters of the nuclei. Dr. Bird has given a table in which the calculi are arranged in classes according to the nature of their nuclei. It fully confirms the generally-received opinion of the great preponderance of uric acid and urates in these deposits as will be seen in the annexed abstract.

*Abstract View of Nuclei.*

| | | |
|---|---|---|
| Nuclei, consisting of | uric acid or urates | 262 |
| ,, ,, | cystic oxide | 11 |
| ,, ,, | oxalate of lime | 45 |
| ,, ,, | phosphates | 21 |
| | | 339 |
| | Mixed calculi | 3 |
| | | 342 |

We may observe that in a subsequent part of the paper the number of calculi containing uric acid or urates as their nuclei, is stated to be 255 instead of 262. How this discrepancy can be explained we do not know. It is also worthy of remark that although the nucleus most commonly occupies the geometric centre of the calculus, this is not always the case; it is sometimes remarkably eccentric, as in certain uniform concretions; and, in a few, several distinct nuclei, or centres of deposition, are met with. In some rare instances the concretion which forms the nucleus is found loose within the body of the entire calculus; a circumstance in all probability arising from a layer of blood or mucus having concreted around the nucleus, and on which the matter forming the body of the calculus became deposited. In this case, on the whole becoming dry, the mucus or blood would be diminished to a very thin layer, and the nucleus detached from the body of the stone. In a few instances calculi appear to possess no nuclei, the centre being occupied by a cavity full of stalactitic or mammillated projections. Dr. Bird has only observed this in uric-acid calculi.

We shall not occupy any time in describing the shape or other external appearances of the various kinds of calculi, and urinary deposits; they have already been frequently noticed in this Journal, and must be familiar to the majority of our readers. But we would particularly direct their attention to the following condensed account of the *microscopic* characters of the different constituents; and that, not only by reason of the intrinsic interest of these observations, but also, and chiefly, because we desire, as much as possible, to urge upon all future inquirers the essential importance of the microscope in almost all investigations into the structure and diseases of the human frame. For the examination of urine, a low power (a good half-inch achromatic object-glass, with a low eyepiece,) is abundantly sufficient. The deposits, after being washed and allowed to dry on a slip of glass, can be beautifully preserved by means of Canada balsam, and thus rendered permanent.

*Uric acid.* We shall not here insist upon the characters of urine containing an excess of this constituent, nor attempt to solve the much litigated question of the state in which it exists in the natural secretion, further than to remark that our author inclines to the opinion of Dr. Prout that ammonia is the solvent. The presence of this salt, the urate of ammonia, can be readily demonstrated, either by exposing the urine to a low temperature, or by evaporating it in the vacuum of an air-pump.

Under the microscope the deposit appears amorphous. Dr. Bird has never met with it in the form of needles, as described by M. Vigla.

Urine containing this deposit occasionally presents a very remarkable phenomenon, after being heated to the boiling point, and kept at that temperature for a few minutes. The salt soon disappears, and is replaced as the ebullition continues, by a deposit which resembles albumen in being totally insoluble in nitric acid : it differs from albumen, however, in the fact that it cannot be precipitated from urine by that acid, and that so high a temperature is required for its development. Its nature is not well understood ; Dr. Bird is inclined to refer it to the same category as the incipient albumen of Dr. Prout.

Uric acid is more rarely deposited in a free state ; it may, however, be thrown down by several acids, and the tint of the crystals will vary according to the amount of chemical action exerted on the colouring matter of the urine by the acid employed. The lightest-coloured crystals are obtained by the use of acetic or phosphoric acid ; those produced by the nitric or hydrochloric are generally deeply tinted, and present, under the microscope, a somewhat complex structure. The crystals are sometimes large enough to be visible by the naked eye. M. Vigla affirms that when the grains of uric acid are as large as this, the deposit is always amorphous. The results of observation in this country are totally opposed to such a statement. The following are the varieties noticed by Dr. Bird.*

A. Rhomboids of a tolerably distinct lozenge-shape. This is the natural form. The acute angles are commonly well defined, but the obtuse corners of the lozenge are often rounded off, so that the whole crystal represents a long elliptical figure with sharp extremities. In the centre there is always a curious angular marking resembling a nucleus.

B. The elliptical lozenge-crystal is sometimes singularly modified. A portion being removed near each acute angle, so that the whole resembles a kind of spindle, sometimes approaching a fleur-de-lys figure. These crystals are often of a beautiful amber colour, and are usually met with in very acid urine.

C. Not unfrequently the outlines of the crystals lose their acute angles, and they then form flat rectangular tables, about twice as long as broad, more rarely they resemble cubes, in which case they generally constitute the whole of the deposit. In the cubes there is evidence of an internal structure ; lines arranged in parallelograms, being readily distinguished by the microscope. If these crystals be allowed to dry, and are then examined after immersion in a fluid, the edges become translucent, and they have the appearance of opaque cubes set in a transparent frame.

D. In very acid urine the long tabular crystals are sometimes seen serrated at their extremities, or so deeply shaded as to give the idea of their being edged with a series of dark compact lines. This appearance is never visible at the sides of the crystal. Under the microscope, these crystals are seen to be intersected by two crescents, the convexities of which are turned towards each other, and sometimes touch.

E. Occasionally the tabular, and more rarely the rhomboïdal varieties are found united in the form of a cross, or stars of various degrees of

* Drawings are given of the most interesting of these and other deposits.

regularity. They are frequent in the bright orange-red sand, and are often abundant in the pink deposits of urate of ammonia.

F. Uric-acid crystals, resembling quadrilateral tables, sometimes, with a carefully-adjusted light, are seen to be composed of a series of short cylinders lying on their sides. The discovery of these belongs to M. Vigla. Some other forms we need not mention.

*Cystic oxide.* The microscope affords the readiest means for the recognition of this rare substance. It is always deposited from the urine in a white crystalline state, and the crystals present themselves under one of the two following forms: 1, Tolerably regular six-sided tables, which are sometimes transparent throughout, but more commonly opaque at the centre; 2, roundish tables, opaque in the centre, and somewhat crenate at the edges. This form has not previously been described by any author. In order to obtain these crystals, the deposit in which they are suspected to exist should be washed with boiling water, to remove urates and any common salt which may be present. The best method to obtain them from a calculus is to digest some of the powder in a warm solution of ammonia, and allow a drop of the clear fluid to evaporate spontaneously on a slip of glass. There is, however, one source of fallacy which must not be overlooked, and this arises from the curious modifications presented by crystals of common salt, when evaporated, either rapidly or slowly from urine; some of these pretty closely resemble the crystals of cystic oxide. They may be detected by the following characters; they are never opaque in the centre as those of cystic oxide generally are; they do not exhibit colours with polarised light, and they are soluble in water.

*Uric (xanthic) oxide.* The account which Dr. Bird gives of this substance is merely an abstract of the observations of MM. Liebig and Wöhler, which have been already partially noticed in this Journal. (Brit. and For. Med. Rev., vol. IX. pp. 364-5.) We shall, therefore, confine ourselves to the transcription of the following tabular views of its characters as contrasted with those of uric acid, with which substance it is most likely to be confounded, the chemical composition of the two being extremely similar:

| *Uric Oxide.* | *Uric Acid.* |
| --- | --- |
| "1. Dissolves slowly, and without any effervescence, in nitric acid. | 1. Dissolves readily, with copious effervescence, in nitric acid. |
| 2. The nitric solution leaves, by careful evaporation, a yellow stain. | 2. The nitric solution leaves, by evaporation, a pink residue. |
| 3. It dissolves in concentrated sulphuric acid; and the addition of water does not render the solution turbid. | 3. It dissolves in concentrated sulphuric acid; and the addition of water produces a copious precipitate of uric acid. |
| 4. Its solution in potass is not precipitated by hydrochlorate of ammonia. | 4. Its solution in potass is precipitated by hydrochlorate of ammonia. |
| 5. It is precipitated pure and free from alkaline combination, when a current of carbonic acid is passed through its solution in potass. | 5. It is precipitated from its solution in potass, by a current of carbonic acid in the form of urate of potass. |
| 6. When decomposed by heat, it does not yield any trace of urea. | 6. Decomposed by heat, it becomes partly converted into urea." (p. 203.) |

The pink colouring matter of urine receives the name of *purpurine* from our author; but neither this, nor the *carbonate of lime*, which is occasionally met with in calculi, need detain us.

We pass on to the consideration of *oxalate of lime*, a most important ingredient of urinary concretions. Dr. Bird is not satisfied with the now commonly received opinion that the oxalic acid is a secondary product of the oxidation of saccharine matter occasionally present in the secretions, and is more inclined to account for its existence by the conversion of uric acid into oxalic acid and urea, as shown in the experiments of Liebig and Wöhler.

The oxalate of lime never occurs in an amorphous form ; it is always regularly crystallized. The crystals are white in colour, sharply defined, and always entire: they are perfectly octohedral, but differ materially in the acuteness of the terminal angles. When these are very acute, the crystals are seen lying on their sides, with their angles and edges exceedingly distinct; when the octohedron is more obtuse, the outline of the crystal appears perfectly square, having another square outline in its centre, so arranged that the angles of the inner square are opposite the sides of the outer one. When these crystals are allowed to dry, and then examined either in a drop of water or Canada balsam, the inner square remains transparent, whilst the outer becomes opaque, and often absolutely black. The shape of the crystals can be well seen by adopting a method pointed out by Mr. S. W. Griffith. Add a few drops of alcohol to an oxalate deposit moistened with water; a series of vortiginous currents, perfectly distinguishable by the microscope, become excited by the mixture of the two fluids; in these the crystals roll over each other, and their true figures become beautifully distinct. Dr. Donné is the only writer who has described the crystals of oxalate of lime, as they occur in urine. It should be observed that the artificial crystals which may be produced by adding oxalate of ammonia to urine, differ considerably from these: they resemble globules, transparent in the centre, and having a marked tendency to become arranged in rouleaus, somewhat after the manner of blood-discs. We must refer to the original paper for a description of the plan by which oxalate of lime may be most easily detected in calculi.

Deposits of *phosphate of lime* are sufficiently common, and the same salt very generally exists in calculi, after forming a considerable portion of their mass. It is, however, exceedingly rare to find it constituting the nucleus. It always occurs in an amorphous state. Such is not the case with the *ammoniacal phosphate of magnesia* (triple phosphate), which presents two distinct forms of crystal, according as the proportion of ammonia varies. The first, or neutral triple phosphate, is frequently met with in the well-known iridescent pellicle which collects upon urine containing this salt, and under the microscope is seen to consist of beautifully defined transparent crystals, all of them being either prisms, or some modification of that figure. They are often strongly shaded on one side, and illuminated at the edges and angles, unless they are immersed in water or some other powerfully refracting medium, when they appear perfectly transparent. The second, or bibasic phosphate, always occurs in alkaline urine, and in all probability is a secondary product. Dr. Bird has never seen it constituting the entire mass of a deposit, but it can frequently be detected in mixture with phosphate of lime. Its crystals consist of thin laminæ, resembling foliage, and sometimes traversed by a transparent cross. When formed artificially by the addition of ammonia to urine, they are always of an elegant stellar shape. When mixed with phosphate of lime they constitute the fusible compound. The nature of any deposit, or fragment of calculus containing phosphates, may therefore be at once determined, by dissolving it in hydrochloric acid, placing a drop under the microscope and adding ammonia; this determines the precipitation, either of an amorphous granular mass, of a series of stellar crystals, or of both together: in the first case, the matter consisted of phosphate of lime; in the second, of the triple phosphate; and in the third, of the fusible compound. A striking example this of the value of such methods of investigation.

We have not room to discuss Dr. Bird's speculations regarding the derivation of these deposits, and as he promises to bring forward the data upon which they are founded at some future period, we abstain altogether from any remarks upon them. The paper, which our readers will see is one of no small value, concludes with a useful table of the *action of re-agents on urine containing deposits*, or as our author somewhat quaintly terms it, at various times in the body of the communication, the *behaviour* of urine under these circumstances.

VII. *On the location of pulmonary phthisis, and its relation to diagnosis, by* H. Marshall Hughes, M.D. This paper comprises the results of numerous investigations into a subject of great importance, and bears throughout the impress of a cautious and discriminating mind. We shall endeavour to present our readers with as complete an abstract as our limits will allow.

Before entering upon the more immediate object of his study, Dr. Hughes makes some remarks upon a form of phthisis, fortunately rare, in which the usual auscultatory signs are absent, the chest being throughout perfectly free from dulness, the voice not abnormally resonant, and nothing being heard by the stethoscope beyond a small mucous or muco-crepitating rattle, universally diffused through every part of the lungs. In such a state of things, it is no marvel that the disease should be mistaken for bronchitis, and the practitioner grievously disappointed at the utter failure of all the ordinary means of treatment. After death, which is seldom long delayed, the mystery is cleared up, and the lungs are found thickly studded with miliary tubercles, or granulations. We have said that a fatal result is, in general, rapidly attained, but such is not always the course of events; the malady may assume a chronic character, and time be given for the softening and suppuration of the tubercles. We extract the following case (abridged,) which not only illustrates this fact, but shows also that the disease may occur in persons who have arrived at or passed the middle period of life. The patient was a male, aged forty-five, and had formerly been intemperate. About a year before he was seen by Dr. Hughes he received a severe blow upon the right side, from the effects of which he believed that he had never recovered. Nine months after this occurrence, he was attacked, by his own report, with inflammation of the lungs, and treated actively. He recovered partially, but the cough did not entirely leave him. When first seen he appeared to be labouring under simple bronchitis and dyspepsia, and was treated accordingly; but after a time "he began to sink rapidly, and though the chest was repeatedly examined, and found uniformly and universally resonant on percussion, gurgling succeeded to simple mucous rattles; and the characteristic sputa of advanced phthisis, independently of other symptoms, indicated but too clearly the nature of the malady. He died about six weeks after I first saw him, and between four and five months after being first troubled with cough. On inspection of the body, twenty-four hours after death, tubercles were found to be equally and universally distributed throughout both lungs. They were in all stages; some were transparent and others opaque; some had become softened, and others, by the suppuration of surrounding parts, had already been expectorated, and had left small cavities of the size of small nuts. There existed no pneumonic consolidation; and the only exception to the general condition of the lungs I have mentioned was a cavity, rather larger than the others, situated in the very apex of the right lung."

We must now follow our author in the examination of the table he has constructed, and which includes the records of 250 cases, 175 being males and 75 females. This table originated from a desire to ascertain which lung was most frequently affected with tubercles; in which, when both were diseased, they were first deposited, or in which they made the most rapid progress; and, what is of far higher importance, in what proportion tubercles were first deposited in the superior lobes of the lungs. A large majority of the individuals were alive at the period of examination; but as cognizance was only taken of those cases in which the disease was in an advanced stage, and where the symptoms rendered the nature of the malady perfectly unequivocal, even without the aid of auscultation, this consideration detracts but little from the value of the deductions. The only exception we would be inclined to make is one which perhaps deserves the name of hypercritical, viz., that as the disease was approaching a termination before the patients were examined, the seat of the *first* deposition could only be ascertained by determining the part most extensively affected, and must therefore rest upon an assumption, probably enough correct, that excavations will soonest occur in the part that was primarily attacked. An example of the table is given, from which the nature of the whole may be readily appreciated.

*Is one lung more obnoxious than the other to the deposition of tubercles?* Laennec believed that the right was most generally affected, and Louis affirms that the reverse is the case. The following table exhibits the results of Dr. Hughes's investigations:

| | | Cases. | per cent. |
|---|---|---|---|
| The left side was chiefly diseased in | | 116 | 46 |
| The right ditto ditto | | 89 | 36 |
| The more diseased side was doubtful in | | 45 | 18 |
| Of the 116 cases on the left side, there were | males | 76 | 43 |
| | females | 40 | 53 |
| Of the 89 cases on the right side, there were | males | 66 | 38 |
| | females | 23 | 30 |
| Of the 45 cases, in which the most diseased side was doubtful, there were | males | 33 | 19 |
| | females | 12 | 16 |

Of the 48 cases examined after death, of which 11 only were females,

| | Males. | Females. |
|---|---|---|
| Tubercles were confined to the left lung in | 3 | 1 |
| " " right " | 1 | 0 |

From the above statements it would appear that the left lung is upon the whole most susceptible of tubercular disease; but the difference is manifestly so slight as to be utterly useless in relation either to diagnosis or prophylaxis.

*In what proportion of cases are tubercles first deposited in the upper part of the lungs?* Of the 250 cases recorded by Dr. Hughes, the upper lobe of one or both lungs was solely or principally diseased in 237 or 95 per cent. Of the remaining 13 cases, of which 11 were males and 2 females, there were 9 or $3\frac{3}{5}$ per cent. of the whole number, in which both lungs were universally and uniformly diseased. Of these nine, 8 were males and 1 was a female. Of the remaining 4 cases, the upper lobe in them was at least equally affected with other parts. In only one case out of the whole 250 were tubercles confined to the base, there being none in the upper lobes of the lungs.

It is not our purpose to enter into any discussion of the diagnosis of phthisis, and we shall therefore abstain from noticing the remarks made by our author in reference to the differential signs between this malady and those with which it is most liable to be confounded; but we must beg leave to refer our readers to the observations of M. Grisolle (noticed in our last Volume, p. 385,) as showing that pneumonia more frequently attacks the upper lobe of the lungs in the first instance than is generally believed in this country; and we shall conclude this abstract by inserting the following table, exhibiting *the age at which phthisis is most fatal*, although the numbers are too limited for certain conclusions to be deduced from it.

"Of persons (not children) who have died, or were in a condition which would soon terminate in death, there were—

| | Males. | per cent. | Females. | per cent. | Total. | per cent. |
|---|---|---|---|---|---|---|
| Under the age of 20 ...... | 14 | 8 | 5 | 7 | 19 | 8 |
| Aged 20, and under 30 ..... | 70 | 41 | 37 | 49 | 107 | 43 |
| „ 30 „ 40...... | 45 | 26 | 21 | 27 | 66 | 26 |
| „ 40 „ 50...... | 39 | 23 | 9 | 12 | 48 | 19 |
| „ 50, and upwards ...... | 7 | 4 | 3 | 4 | 10 | 4 |
| Total ............ | 175 | | 75 | | 250 | |

(p. 258.)

viii. *On the proceeding to be adopted in a case of injured intestine, from a blow upon a hernial sac, by* C. Aston Key, Esq. Mr. Key recognizes three states of the bowel which may be produced by an accident of this nature: the coats may become merely inflamed in consequence of the contusion, or the violence may be such as at once to rupture the intestine, or, failing this, the contusion may be so severe as to be followed by sloughing and escape of fæces. The treatment of each of these must necessarily vary. In the first case, the contents of the sac should be returned and the ordinary antiphlogistic regimen adopted, with this exception, that purgatives should be most carefully abstained from. We think Mr. Key is perfectly correct in dwelling with much emphasis on this point. When there is either inflammation or a tendency to that state in any other organ of the body, our attention is at once drawn to the necessity of keeping it at rest for some time; why should we pursue a different course when the intestinal canal is the suffering part? It would be hard to find any better reason than common routine habit. In the severer forms more active proceedings are required, and the safety of the patient will depend upon a free exit being afforded to the offending matters. The sac, therefore, should be at once laid open (i. e. whenever symptoms of rupture present themselves,) and a temporary artificial anus established. The cases related are interesting and instructive.

IX. Mr. France has related a case of *irideremia*, or *absence of the iris*, an exceedingly rare malformation, in this instance apparently congenital.

X. Dr. Babington records *a case of enormously distended gall-bladder*. The cyst contained at least three wash-hand basins of fluid. The nature of the case was not suspected during life, and treatment was of course of no avail.

XI. *On pneumonia, by* H. M. Hughes, M.D. This paper is intended to form a sequel to the one on phthisis noticed above. It is not devoid of interest, but the absence of anything peculiarly novel in the views presented, renders a detailed analysis unnecessary, seeing that the disease in question has formed the subject of such frequent discussion already in the pages of this Journal. The results are drawn from the records of cases observed in Guy's Hospital, and which are arranged in two tables; the one consisting of cases of primary pneumonia; the other of cases in which distinct evidence of the disease was found after death, the patients having been, for the most part, the subjects of other and more chronic affections. Our attention in reference to both must be confined to the subject of "location." In the *first* table we have the account of 101 cases. In 19 of these *both* lungs were diseased; in 29 the *left* alone was affected; while the *right* was involved in 52. The disease was confined to the *base* in 62 cases; it occupied the *whole lung* in 12; it was limited to the *posterior* parts of the organ in 8; to the *apex* in 5; and to the *centre* in 3. It was situated in various parts of one or both lungs in 9; and in 2 cases the locality was not mentioned.

The second table contains the records of 145 cases, in 60 of which *both* lungs were affected; in 43 the disease was limited to the *right*, and in 40 to the *left* lung. The parts principally or solely involved were as follows: the *upper lobe* in 13, the *centre* in 7, the *posterior* in 4, and the *base* in 49. The whole organ was generally diseased in 65.

We should scarcely have noticed these results, but for the sake of comparison with the statistics given in the former paper; our readers will see, at once, that in this point of view they have considerable value, the contrast in the allocation of the two diseases being of so striking a nature.

XII. *Cases of hemorrhage after delivery, by* Dr. C. W. Lever. The author's object is to call attention to two predisposing causes of hemorrhage, which are not generally recognized by obstetric writers, viz., diseases of the spleen and kidneys. In reference to the *first*, he has arrived at the following conclusions: "1. That in females affected with enlargement or disease of the spleen, the uterus is predisposed to dilate, and therefore admits of the effusion of blood into its cavity. 2. That the blood so collected coagulates, and excites considerable irritation, as marked by the accession of rigors, fever, &c. 3. That the fever so produced, in course of time, (varying in different cases,) assumes the intermittent type, especially when the patients have previously suffered from ague. And 4. That such intermittent fever is curable by the same remedies as are successful in the treatment of pure and uncomplicated ague." In regard to the *second* cause, he believes: "1. That labour occurring in patients affected with morbus Brightii is generally lingering. 2. That in such patients, although the fœtus and its secundines may be expelled by the natural uterine efforts, and the uterus may for a time appear to contract, yet that it is very liable to become relaxed, and distended with blood 3. That in patients so affected, peritonitis of a more or less acute character is prone to occur." We recommend this paper to the careful notice of our obstetric brethren, whose attention cannot be too forcibly directed to the distressing occurrences of which it treats.

XIII. *On the microscopic globules found in urine, by* Dr. Golding Bird. The author distinguishes four kinds. 1. *The true pus-particle,* roughly granular on its surface and evidently compound in its structure, becoming reduced on the addition of ammonia to a mucous mass, readily miscible with water; in which, however, the particles may be discovered, apparently shrivelled. These particles are partially dissolved by acetic acid, leaving numerous minute transparent circular bodies, which are regarded as their nuclei; they are free from granulations, and about one fourth the size of a blood-disc, or 1-8000th of an inch in diameter. The urine is always albuminous, and becomes opaque on the application of heat.

2. *The true mucous globule,* generally rather smaller than the pus-particle, being about 1-2000th of an inch in diameter, appearing circular, with a smooth well-defined edge, under a low magnifying power, but becoming distinctly granular on the surface with a power of 250 diameters. They can be distinguished from the former only by their being not readily miscible with water, becoming less shrivelled on the addition of ammonia, and having fewer and less distinct granulations. They are to be found in all urine after it has been allowed to stand for some time, and are increased in quantity when there is irritability of any part of the urinary mucous membrane. When this last condition co-exists with diuresis or a frequent desire to pass urine, they become replaced by the third species, or,

3. *The large organic globule,* which varies in size from 1-1000th to 3-1000th of an inch in diameter, and is with great difficulty distinguished from the pus-particle. These globules rarely, if ever, constitute a deposit, but are found free and floating in the urine, generally scattered and often few in number. They are not so evidently compound as the pus-globule, though, when treated with acetic acid, they become broken up, and have similar minute transparent globules. Dr. Bird thinks this variety is identical with that described by Rayer under the name of muco-pus. These globules are abundant in the urine of pregnant women, especially during the latter months, and when there is frequent desire to empty the bladder. Dr. Bird has also found them in every case of ardor urinæ which he has examined, and also in the urine of patients labouring under Bright's disease. He has detected them also in several cases of malignant disease of the womb.

4. *The small organic globules.* These are minute and very rare. They are apparently perfectly simple in their structure, absolutely spherical, free from granulations, and about 1-3000th of an inch in diameter. They mix with water with the utmost readiness, and undergo no change whatever. They often form a glistening white deposit at the bottom of the capsule in which the urine has been allowed to cool after being gently heated; and in this state closely resemble, to the naked eye, the oxalate of lime deposit. Dr. Bird has only met with this variety in two cases, and in each its appearance was evanescent: both specimens of urine were passed by women during menstruation.

xiv. *Case of poisoning by arsenic, by* Mr. Hilton. The value of this paper consists in the admirable analysis conducted by Mr. A. S. Taylor. It is impossible to draw up anything like a perfect abstract, and we must be contented with noting one or two points of special interest. A man aged thirty five swallowed, by his own confession, upwards of a quarter of an ounce of arsenic, from the effects of which he died in ten hours. The stomach was highly inflamed, especially at the pyloric extremity; it contained about a pint of dark-brown mucous fluid, in which some very small granules of the poison were mixed; and its coats were lined with a large quantity of semi-transparent glairy adhesive mucus, which included numerous portions of arsenic. Though there was enough arsenic in the stomach to saturate the contained liquid fully, yet, when the solid matters were allowed to subside, *not a trace* of the poison could be detected in it. We need scarcely point out the lesson which this fact should teach. Four ounces of the blood were examined, according to the improved method recommended by Orfila, but without success, probably on account of the small quantity employed. Mr. Taylor recommends sulphuric acid as a carbonizing agent, in preference to the deflagrating action of nitre. The spleen, analysed after the manner pointed out by MM. Danger and Flandin, gave equally negative results; but when seven ounces of the liver were treated in the same way, slight traces of the presence of the poison were clearly detected. We strongly recommend a perusal of the original.

xv. *Case of fatal pleuritis, by* Mr. W. G. Carpenter. This is a very singular case. On examination after death, the right pleural cavity was found to contain a large quantity of sero-purulent fluid, and "*a piece of ivory* worked into four artificial front teeth, covered with a brownish crust, with a pointed piece of silver rivetted into the upper part of the teeth, which had evidently assisted in fixing them to the upper jaw; the base of the silver rivet surrounded with wadding." There was an old fistulous opening on the outer surface of the lung, large enough to admit the tip of the little finger. Upon inquiry, it was discovered that the patient, who had been afflicted with asthmatic bronchitis from childhood, had "swallowed" the teeth, during a fit of coughing, thirteen years before his death.

xvi. ***Observations on inflammation of the aqueous membrane of the***

*eye, by* Mr. S. R. Bedford. *Aquo-capsulitis,* or inflammation of the aqueous membrane of the eye, it is stated, is essentially marked by the appearance of a general or partial opalescence, of various degrees of intensity, and which may have its origin in the corneal, iridal, or capsular portion of the structure. When seated on the corneal part, it may be mistaken for corneitis, or corneal conjunctivitis; but a careful examination of the organ, in different lights, will generally lead to a correct diagnosis. There is greater difficulty in determining the true nature of the case, when the iridal portion is affected; but this is practically of little moment. The opalescence does not appear to depend upon morbid deposition, but is simply the effect of vascular turgescence, occurring in a transparent membrane. The disease may terminate in increased secretion, in fibrinous or puriform effusion, and in ulceration. 1. The *first* result, or, in other words, dropsy of the anterior chamber, is well known as a most obstinate affection. Mr. Bedford thinks that evacuation of the aqueous humour is a measure of very doubtful value, as far as a radical cure is concerned, and should only be adopted when the pain, caused by the distention, is extreme. He narrates some cases which go to prove that it may be relieved, at least, by ordinary means of treatment. 2. *Fibrinous effusion,* in the first stage, is amenable to simple remedies; and, when more advanced, yields to slight mercurial action. The membrane, in this state, has sometimes a peculiar spotted appearance, which Mr. Bedford regards as pathognomonic of the disease. The spots are distinctly visible, very circular, and uniformly diffused. 3. *Puriform effusion* is less common. When it exists to a great extent, evacuation may be resorted to with advantage: but in most cases the matter is absorbed, under the action of the ordinary means of cure. 4. *Ulceration* is the least frequent termination.

We can conscientiously recommend the whole paper to the consideration of all who are interested in the diseases of the eye. It is drawn up with much care, and the cases are narrated with a precision and minuteness that are worthy of all praise: but we were somewhat surprised to find no mention of any local applications, in the cases of ulceration of the cornea which are related. The value of a strong solution of the nitrate of silver in such cases is too well known to require a defence here.

xvii. *Observations on the diseases of the orifice and valves of the aorta, by* Norman Chevers, m.d. This is an exceedingly valuable contribution to pathology, and fully maintains the author's character for elaborate minuteness of research. We shall present our readers with as complete an analysis as our limited space will allow.

Great difference of opinion still exists in reference to the nature, the causes, and the effects of the various organic changes which are so frequently observed at the commencement of the aorta; and much of this appears to arise from the common error of attributing to disease, and of charging with the production of morbid symptoms, many slight deviations, which are either unimportant in themselves, or are the results of means adopted by nature, either to limit the extension or repair the ravages of a destructive process, or to adapt the uninjured portions of a diseased heart to the new circumstances in which they are to act. The object of the paper before us is to place the question upon a more satisfactory basis, from the examination of a large number of aortæ both in a state of health and disease.

*Dilatation of the aortic orifice.* In order to arrive at an accurate knowledge of the dimensions of this part, it is necessary to measure the circumference of the vessel both above and below the valves, because, even in the healthy state, there is a considerable difference between the two, and this disproportion is greatly increased and varied in every case of organic disease of the heart and arteries. The measurement of eight healthy aortæ in these situations gave the following results. The mean circumference of the canal immediately below the valves was thirty-six and a half lines ; that precisely above, thirty-four lines. But upon traction being made, (after separating the vessel from the heart,) the latter portion was found to be dilatable to forty-three lines, the former only to thirty-nine and a half; proving, that while the lower part of the ostium is the wider, the upper is by far the most dilatable.

*Dilatation of the lower part of the orifice* becomes developed to a greater or less extent in nearly all cases of eccentric hypertrophy of the left ventricle; it also frequently, but not always, accompanies simple dilatation of that cavity. The most common immediate cause is, either thickening and contraction of the aorta above or behind the valves, or disease of the sigmoid crescents themselves interfering with the free emptying of the ventricle.

*Dilatation of the superior part of the ostium* usually results from obstruction existing either in the main trunk, or in some of the terminal branches of the aorta. It is generally found in cases of thoracic and abdominal aneurism; in stricture of the descending arch, where the ductus arteriosus is closed; in association with extensive ossification of the smaller arteries; and in the subjects of chronic renal and hepatic disease. This part yields first, because it is placed between two forces, viz., the active impelling power of the ventricle, and the fixed resistance of the obstacle, and because it is in itself much more dilatable than the lower portion. The ventricle does not begin to suffer undue dilatation until the whole of the vessels between the heart and the impediment have been stretched to the uttermost. Previously, therefore, to this occurrence we have expansion of the upper part of the orifice, with a smaller state of the lower, but without actual contraction of this part; for the ventricle, though thickened in its walls, does not become altered in its cavity for some time.

When this state of parts exists, i.e. when the upper portion of the aortic ostium is more dilated than the lower, the most usual condition of the valves is, either great lengthening of their marginal cords, or depression of their superior points of attachment, with shallowing of the pouches, and rounding or, occasionally, more or less retroversion of their free borders. But, in many cases, Dr. Chevers has observed a singular natural adaptation of the parts, by which the ill effects of regurgitation were obviated : " the upper margins of the sigmoid curtains having been stretched laterally, their crescents, even though rendered rather shallower than natural, will still perform their valvular office ; as, having lost their usual horizontal position, they bag down into the still undilated opening of the lower part of the ostium, and, their edges there coming into opposition, the vessel is perfectly closed against the reflux of blood into the ventricle." It is probable that the good effects of this arrangement are but of temporary duration, for as the edges of the valves only are in contact, there is a great tendency for the edge of one to be pressed below the others, or retroverted by the force of the descending blood at each ventricular diastole. It is clear, therefore, that the rule laid down by some authorities, that dilatation of the orifice of the aorta must of necessity be attended by inefficiency of the valves admits of some qualification. Even when both parts of the ostium are largely dilated, the valves, if undiseased themselves, may perform their office, for they become widened and deepened in proportion to the expansion of the tube.

When the valves are in the position above noticed, viz., in contact by their edges only, they are also very apt to become sacculated below, and are thus exposed to the danger of sudden rupturing, or of more gradual perforation from the ulceration of atheromatous or other matter deposited in their structures. And this leads us to the next point in our inquiry.

*Rupture and ulceration of the sigmoid valves.* In sixteen cases out of nineteen of this nature, examined by Dr. Chevers, the lower part of the aortic ostium was morbidly wider than the upper. In eleven of these, the upper was much contracted, while the lower was greatly too wide; and in the other five, the upper was rather widened, but less so in proportion than the lower. In the seventeenth and eighteenth, the superior was dilated, while the lower was rather contracted; and in the last, the whole of the orifice was far too narrow; but in this case, another part of aorta was congenitally malformed. It becomes here an interesting subject of inquiry, whether the lesion of the valves was the cause or the effect of the dilatation of the orifice. Dr. C. inclines to the latter opinion, seeing that in nearly two thirds of the cases, there was quite enough to account for the dilatation, altogether independent of the state of the valves. We think the question is still open to investigation. The edges of these lacerations are often covered with granular masses of coagula, which of course tend to lessen the amount of blood which regurgitates. Our author has observed also, "that where a lacerated curtain has become much loaded with fibrinous or boney concretions, the sinus behind is usually far deeper than the other two; as if this indentation allowed the thickened mass as much room as possible to recede while the blood is passing into the aorta." Such a state of disease may continue for some time without proving fatal; of this fact Dr. Chevers relates a very striking instance. (p. 404.)

*Atrophy of the semilunar valves.* This state of disease our author believes to be exceedingly rare; at least, unless this term be applied to those cases in which, from morbid depositions between the laminæ, the nourishment of the parts has become defective. The cribriform state of the valves, which is occasionally observed, the edges of each opening being quite smooth and covered by a reflection of the serous membrane, and which has been attributed to the same cause, he conceives to be congenital, depending merely upon an arrest of development during an early period of intra-uterine life; for, among other reasons, he has observed it three times in the aorta of children about four years old, and once in an infant which died at, or very shortly after, birth.

*Vegetations.* Two kinds of these are observed: granular clots, evidently mere deposits of fibrin from the blood, and minute semi-transparent warty bodies, which some authors regard as of a similar nature. With respect to these latter, Dr. Chevers has been led to form a different opinion. He regards them as duly organized growths, seeing that their form is unvarying and symmetrical; that they are completely blended in with the serous membrane, and that he has found them assuming a deep yellow hue in jaundice; and he believes that they are intended to *retard* the progress of diseased actions in the parts upon which they appear.

*Contraction of the aortic orifice.* This may occur in three situations, viz. either below, above, or within the curtains, the first of which is the most rare. This state is generally occasioned by local disease in the tissues; but there are several classes of cases in which, without any evidence of such lesion, the whole of the aortic trunk with its appendages is found unusually small. Upon this point Dr. Chevers has recorded some valuable observations, for which we must be contented with a simple reference to the original; and thus conclude our imperfect sketch of what we cannot but characterize as a most interesting production.

xviii. *Case of contracted aorta, by* W. Muriel, Esq. The constriction, which almost amounted to obliteration, was near the junction of the *ductus arteriosus*: the superior intercostals were enormously enlarged.

xix. *Two cases of disease of the larynx, requiring laryngotomy, by* Mr. Hilton. These cases deserve perusal, but are not susceptible of condensation. The operation, in one case, was performed by means of a curved trocar and canula, which Mr. Hilton thinks preferable to the knife.

xx. *On the operation for cataract, by* Mr. Morgan. The peculiarities of Mr. Morgan's methods of proceeding are as follows: the needle is extremely fine, of the same thickness from the point to the handle; cutting at the point but not at the sides, and hardly longer than the diameter of the globe. It is passed through the sclerotic, at the distance of rather more than a line from the cornea, and just below the transverse diameter; it is then carried, with a slight inclination forwards, directly through the central substance of the cataract, completely transfixing the lens. This requires to be done with much care: the needle should be *drilled* through, by rotating the handle of the instrument between the finger and thumb. The lens is then *pulled* out of its place with great ease and precision; and when it has reached the situation it is intended to occupy, the needle is cautiously withdrawn by a similar rotatory motion.

xxi. *Observations on certain diseases originating in early youth, by* Dr. G. H. Barlow. The publication of this paper enables us to perform a promise made some quarters back to our readers, by reviewing a communication of the same author, which we omitted in our notice of the 13th number of the Reports. The two memoirs, taken in connexion, form an essay of much interest and great value, upon a subject of the highest importance. All the cases narrated have reference to diseases of the circulating and respiratory organs, and may be resolved into four classes: 1. Those in which obstruction to the circulation in the right side of the heart was produced simply by defective expansion of the lungs and air-passages. 2. Those in which it was the result of pericarditis acting *mediately through the impediment opposed to the respiratory movements.* 3. Those in which it resulted directly from the obstruction to the return of the blood to the left side of the heart, produced by contraction of the left auriculo-ventricular orifice. 4. Those in which the origin of the mischief was of a more complicated nature, depending upon two of these causes conjointly, or upon all combined. Our limited space prevents our entering upon a full consideration of the whole; and we shall therefore, in a great measure, confine our notice to the first class, in reference to which the author's views appear to us to possess the most originality.

Under this head we have the details of two cases. The symptoms in each were nearly similar, viz. dyspnea commencing at an early age, disordered circulation, and dropsy, proving at length fatal. The one, a girl, died at the age of sixteen; the other, a male, at the age of eighteen; and in neither were there any signs of puberty. In the first case, that of the female, the right side of the heart was enormously enlarged, both by hypertrophy and dilatation, and the tricuspid valve inadequate to close the auriculo-ventricular opening: the pulmonary artery was smaller than natural, but healthy. The left side was also enlarged, but not to the same extent; the aortic valves healthy, and also the vessel, which however was too small. The trachea was small; there was an obvious narrowing about an inch and a half above the bifurcation, and both bronchi were compressed and flattened. The chest was narrow and contracted, and the lungs fleshy. The liver was very much enlarged. In the second case, the alterations in the heart were very similar; enlargement of the right side, and contraction of the orifices of the pulmonary artery and aorta, both vessels being smaller than natural; but there was also atheromatous matter under the lining membrane of the pulmonary artery, throughout its ramifications. The left bronchus was much flattened, and the trachea and larynx so small that they might have belonged to a boy of six years old. The liver was large, dark, and hard.

In both these cases Dr. Barlow regards the *narrowing of the trachea* as the true cause to which all the subsequent phenomena are to be attributed, the series of causation being as follows: "About the age of eleven in the first case, and fourteen in the second, growth, and the consequent increase in the quantity of the blood returned directly by the cavæ, called for a corresponding increase in the respiratory function. This, however, could not be effected from the imperfectly expanded state of the lungs, arising most probably, as has been shown, from the narrowness of the trachea. The immediate effects of this were, diminished flow of blood from the right ventricle, and narrowing of the pulmonary arteries. Hence arose the distention and hypertrophy of the right ventricle. The accumulation of blood in this ventricle caused either regurgitation into the right auricle, or opposed such an obstacle to the exit of the blood from thence, that it became, in its turn, greatly distended, and the distention being kept up by the pressure of the blood in the veins, dilatation and hypertrophy of the auricle ensued; the concomitant dilatation of both ventricle and auricle giving rise to the enlargement of the auriculo-ventricular opening, and inadequacy of the tricuspid valve to close that opening. The immediate effect of this state of things would be the accumulation of blood in these reservoirs, if I may so term them, the liver and the spleen; and the secondary consequence, accumulation in the whole venous system, giving rise to the lividity and anasarca observed in both cases. The same cause must also have impeded the passage of the blood through the venæ cavæ hepaticæ, and consequently through the portal veins, and thereby have given rise to the ascites."

The application of these views, thus lucidly stated, to treatment, both in the way of prevention and cure, is sufficiently obvious, and need not detain us; but we cannot pass them by without expressing our admiration of the philosophic manner in which our author addresses himself to the often difficult task of analysing complicated symptoms, and reducing them to a simple and intelligible form. Would that the same qualities were more common!

The *second* class of cases, viz. those in which the cardiac disease was the result of pericarditis acting mediately through the impediment which it opposed to the respiratory movements, is well illustrated by an interesting case, (No. vi.) to which we must refer our readers. We beg their particular attention to it, because the influence of this disease in preventing the due expansion of the lungs is, we apprehend, not generally recognized, and observers are too apt to ascribe all the changes detected in the heart to it alone, as a *primary* cause. We would remark, also, that pericarditis occurring in a subject whose growth is completed, does not, when the valves are unaffected, lead at once to hypertrophy, as is commonly supposed, but tends rather, in the first place, to produce diminution in the volume of the heart. This point is noticed and well illustrated by Dr. Chevers in the paper examined above, (vide p. 421, et seq.) The third and fourth classes do not require any observations on our part. We earnestly recommend a careful perusal of the two memoirs.

---

